# Scoping out the literature on mobile needle and syringe programs—review of service delivery and client characteristics, operation, utilization, referrals, and impact

**DOI:** 10.1186/s12954-018-0212-3

**Published:** 2018-02-08

**Authors:** Carol Strike, Miroslav Miskovic

**Affiliations:** Dalla Lana School of Public Health, University of Toronto, Toronto, ON M5T3M7 Canada

**Keywords:** Needle and syringe programs, Mobile service, HIV prevention, Scoping study

## Abstract

**Background:**

Needle and syringe program (NSP) service delivery models encompass fixed sites, mobile services, vending machines, pharmacies, peer NSPs, street outreach, and inter-organizational agreements to add NSP services to other programs. For programs seeking to implement or improve mobile services, access to a synthesis of the evidence related to mobile services is beneficial, but lacking.

**Methods:**

We used a scoping study method to search MEDLINE, PSYCHInfo, Embase, Scopus, and Sociological for relevant literature. We identified 39 relevant manuscripts published between 1975 and November 2017 after removing duplicates and non-relevant manuscripts from the 1313 identified by the search.

**Results:**

Charting of the data showed that these publications reported findings related to the service delivery model characteristics, client characteristics, service utilization, specialized interventions offered on mobile NSPs, linking clients to other services, and impact on injection risk behaviors. Mobile NSPs are implemented in high-, medium-, and low-income countries; provide equipment distribution and many other harm reduction services; face limitations to service complement, confidentiality, and duration of interactions imposed by physical space; adapt to changes in locations and types of drug use; attract people who engage in high-risk/intensity injection behavior and who are often not reached by other service models; and may lead to reduced injection-related risks.

**Discussion:**

It is not clear from the literature reviewed, what are, or if there are, a “core and essential” complement of services that mobile NSPs should offer. Decisions about service complement for mobile NSPs need to be made in relation to the context and also other available services. Reports of client visits to mobile NSP provide a picture of the volume and frequency of utilization but are difficult to compare given varied measures and reference periods.

**Conclusion:**

Mobile NSPs have an important role to play in improving HIV and HCV prevention efforts across the world. However, more work is needed to create clearer assessment metrics and to improve access to NSP services across the world.

## Background

A large body of evidence supports the effectiveness of needle and syringe programs (NSPs) to reduce injection risk behaviors that can lead to infections such as human immunodeficiency virus (HIV), hepatitis C (HCV), and other drug-related problems [1–5]. Over time, NSP service delivery models have expanded to encompass fixed sites, mobile services, vending machines, pharmacies, peer NSPs, street outreach, and inter-organizational agreements to add NSP services to other programs (e.g., hospitals, homeless shelters) [6–10]. The varied models of NSP service delivery are designed to improve access for people who use drugs in terms of time of day, geographic location, and service complement. In their guidelines, WHO, UNAIDS, and UNODC [11] recommend the use of mobile NSP units as an alternative and complementary source for delivery of NSP and other harm reduction services.

For programs seeking to implement or improve mobile services, access to a synthesis of the evidence related to mobile services is beneficial, but lacking. Systematic reviews of NSP-related evidence have been published but none provide an in-depth focus on the operational issues specific to mobile NSPs. Indeed, a review of reviews by Macarthur et al. found no reviews that examined the impact of mobile NSP on injection risk behaviors [3]. As programs attempt to break through the plateau reached in the prevention of injection-related blood-borne infections [12, 13] through service improvements, access to such a review can be helpful for decision-making and service design. We used a scoping study, a rigorous and comprehensive method [14], to identify and describe literature about mobile NSPs and specifically to answer this question: what is known about the design, operational characteristics, and services offered, client characteristics, service utilization, and impact of mobile NSPs? This method allowed for a broad charting of literature without narrow restrictions (e.g., specific populations, specific types of study designs, and/or highly specific definitions of methodological rigor) that might reduce what can be learned from existing literature [14, 15].

## Methods

Using the methods put forth by Arksey and O’Malley [14], our scoping study was conducted in five stages: identify the research question, identify relevant studies, select the studies, chart the study data and collate, and summarize and report the results. The search strategy was designed to identify studies published before November 2017 that addressed any of the following: (1) the design, operational characteristics, and services offered, (2) client characteristics, (3) utilization, and (4) impact. We searched the following databases: MEDLINE, PSYCHInfo, Embase, Scopus, and Sociological abstracts using the following keywords:

- (needle* or syringe* or NSP or NEP or NEX) and (program* or exchange or outreach or distribution) and (mobile or van or bus);
- harm reduction and (program* or outreach or prevent*) and (mobile or van or bus)
- harm reduction and (program* or outreach or prevent*) and (mobile or van or bus)
- harm reduction and (program* or outreach or prevent*) and (mobile or van or bus)
- (needle* or syringe* or pipe* or NSP* or NEX or NEP*) and (crack or cocaine or meth or amphetamine or metamphetamine) and (HIV or HCV) and (mobile or van or bus)
- (needle* or syringe* or pipe* or NSP* or NEX or NEP*) and (crack or cocaine or meth or amphetamine or metamphetamine) and (HIV or HCV) and (mobile or van or bus).

Since our focus was on the mobile programs who offered the distribution of sterile injecting supplies as a main or a part of the services they offered, we excluded manuscripts about mobile programs that provided harm reduction services (e.g., substitution treatment, condom distribution, referrals, counseling, safer use leaflets, HAART treatment, HIV/HCV testing) but did not offer NSP services. We also excluded manuscripts that reported the overall effectiveness of the NSP programs without specifically identifying a role of the mobile services, or lacked empirical data. We also excluded articles that pertained to mobile methadone maintenance treatment (MMT) or mobile-supervised injection sites unless they also provided needle and syringe distribution. Since we did not have resources to translate manuscripts, we excluded articles written in a language other than English. From this process, we identified 1313 manuscripts and after removing duplicates were left with 706. We reviewed the full abstract for each manuscript and removed those that did not meet the eligibility criteria, which reduced the total number to 35 articles. Many manuscripts were excluded because empirical data were not reported. We identified another four relevant manuscripts from the reference lists of the included articles. Next, we searched Google Scholar using the same combination of keywords to find any additional manuscripts that were not captured using database searches. The Google search yielded 108 results of which eight met our inclusion criteria and had not been captured by previous searches.

Based on the extraction tool proposed by Arksley and O’Malley [14], we created an extraction tool specific to our scoping study to capture and organize data relevant to our research question. Data extracted from the studies were divided in five categories: (1) author/year, (2) study design, (3) methods and recruitment, (4) short description of the study/objectives, and (5) outcomes/findings. The extraction tool we used allowed us to “chart” the data—to identify what types of studies are available and what is the extent of the research on mobile NSPs and the main areas of interest. After the data were extracted and reviewed, three further articles were excluded from the study because the data presented were not relevant to our research question.

## Results

Database and Google searches, as well as review of manuscript reference lists, yielded 43 relevant manuscripts with publication dates ranging from 1975 to November 2017 (see Table 1). Charting of the data showed that these publications reported findings related to the service delivery model characteristics, client characteristics, service utilization, specialized interventions offered on mobile NSPs, linking clients to other services, and impact on injection risk behaviors.

**Table 1:** Summary of included studies

Author/year	Study design	Methods and recruitment	Study description	Outcomes/findings
Allen et al. 2015 (USA)	Cross-sectional	Analysis of data collected as part of a PWID population estimation study	To examine differences in access to SEPs between current and former PWID seeking services at a mobile SEP in Washington, DC	The independent samples *t* test showed that the difference in mean walking distance between active and former PWID was statistically significant (*p* < .05), with active PWID having a mean walking distance of 2.75 miles and former PWID having a mean walking distance of 1.80 miles. The results of this study suggest that former PWID who are engaging with SEPs primarily for non-needle exchange services (e.g., medical or social services) may have decreased access to SEPs than their counterparts who are active injectors.
Altice et al. 2003 (USA)	Cohort	*N* = 13 A pilot project: HIV therapy was offered to 13 CHCV clients who met the following criteria: (1) confirmed HIV status; (2) active use of heroin; (3) eligibility for antiretroviral therapy	A pilot project among out-of-drug treatment IDUs infected with human immunodeficiency virus (HIV); HIV therapy was successfully provided to active heroin injectors using the Community Health Care Van (CHCV) at sites of needle exchange	The New Haven NEP provides clean syringes and paraphernalia to approximately 250–300 unique clients monthly. This small pilot study suggests that health services based on needle exchange may enhance access to HAART among out-of-treatment HIV-infected IDUs. In addition, it demonstrates that this population can benefit from this therapy with the support of a nontraditional, community-based health intervention.
Bowser et al. 2010 (USA)	Cross-sectional	From January 2002 to May 2006, 487 unduplicated clients were recruited in year-long cohorts and offered services (*n* = 487)	Examining the impact of MORE (mobile outreach drug abuse prevention and HIV harm reduction program primarily for ex-offenders who are active drug users) on reduction of drug use and re-incarceration for drug-related crimes	By the 6 and 12-month follow-up interviews, active drug using clients reported significant reductions in their use of alcohol, cocaine/crack, heroin, and fewer sex partners and crimes. Program completers reported significantly reduced cocaine/crack and heroin use as well as fewer days in jail and crimes than non-completers (*p* < .01 to .001).
Courty 1999 (France)	Case study		Describing the setting up of a mobile needle exchange bus, its functioning and the resulting coordination with the various partners	The people attending the Bus are predominantly young people under the age of 18 years, who declare their limited information concerning the various health problems. They acknowledge that they do not attend the usual institutional structures, which fail to recognize them. The exchange-prevention bus therefore plays a role in access to information, care and various services as an intermediary between the young person’s demand and conventional institutions. By providing a structure for exchange on drug addiction and AIDS, the Bus creates a first link between users and institutions, but also a link between users of the Bus, which is the first step towards a social link different from that usually generated by addictive behavior.
Deering et al. 2011 (Canada)	Cross-sectional	A detailed questionnaire was administered at baseline and bi-annual follow-up visits over 18 months (2006–2008) to 242 FSWs in Vancouver, Canada	Examining the determinants of using a peer-led mobile outreach program among a sample of street-based female sex workers (FSWs) who use drugs and evaluate a relationship between program exposure and utilizing addiction treatment services	In 2006, an average of 1496 women accessed the MAP van per month, and 1432 condom packs and 3241 clean needles were distributed per month Female sex workers at higher risk for sexually transmitted infections and violence are more likely to access this peer-led mobile outreach program and suggest that the program plays a critical role in facilitating utilization of detoxification and residential drug treatment.
Heimer 2008 (USA)	Cross-sectional	*n* = 1843 New Haven; *n* = 1022 Chicago; active drug injectors; Analysis of program tracking data	Comparing two mobile syringe exchange programs operated with very different exchange policies	Mobile van in New Haven—100,000 syringes distributed between November 1990 and October 1993. Data collected from two convenience samples of 1523 injectors between 1990 and 1993 and from 320 injectors between 1999 and 2001 found mean injections per month of 87 and 82, respectively. Mobile van in Chicago—Data collected from two independent convenience samples of 733 injectors between 1997 and 2000 and from 289 injectors between 1998 and 2000 both found mean injections per month of 75.
Hyshka et al. 2012 (Canada)	Review	A review of 15 years of research on needle exchange in Vancouver’s DTES	Demonstrating that: (1) NEP attendance is not causally associated with HIV infection, (2) frequent attendees of Vancouver’s NEP have higher risk profiles which explain their increased risk of HIV seroconversion, and (3) a number of policy concerns, as well as the high prevalence of cocaine injecting contributed to the failure of the NEP to prevent the outbreak	NEP mobile vans would visit areas with a high prevalence of injection drug use during the evening hours to supplement the fixed site. Demand for syringes increased significantly (when cocaine became the main drug) and the Vancouver NEP limits were doubled from 2 syringes to 4 syringes per day or 28 per week (3 per mobile van visit), and a second van was added in 1993. In response to rising HIV prevalence rates, public health officials increased the NEP budget significantly and by 1995 a third van had been added and the exchange limits were again doubled. Despite often being the only source for sterile syringes during the evening hours, many IDUs experienced trouble meeting up with the exchange van or spent their time in areas not covered by the fixed and mobile NEP sites.
Islam and Conigrave 2007 (Australia)	Review	A literature search revealed 40 papers/reports, of which 18 were on dispensing machines (including vending and exchange machines) and 22 on mobile vans	The aim of this review is to examine, based upon the available international experience, the effectiveness of syringe vending machines and mobile van/bus based NSPs in making services more accessible to these hard-to-reach and high-risk groups of IDUs	Services through dispensing machines and mobile vans have been reported to be responsive to a wider range of IDUs and most importantly to hidden and harder-to-reach IDUs in the community, who for several reasons do not or cannot attend conventional NSPs. Unlike dispensing machines, mobile vans do not provide completely anonymous access to sterile injecting equipment, but peer staffed mobile vans can render a congenial environment that provides near anonymous access. Mobile vans can cover a greater geographic area and can more readily accommodate changes in local conditions. A van of this sort generally follows a relatively consistent route, and parks at a predictable location at a predictable time, although it can change in response to immediate neighborhoods conditions (e.g., increased police presence) or to incorporate additional populations of injecting drug users. One van may visit multiple sites in a single outing. It can provide the benefits of both a fixed and a mobile site. In addition, it can also provide shelter and some security for staff, some privacy for clients, and a consistent service while covering a large geographic area. A roving site also keeps staff members and clients relatively inconspicuous to neighbors, local business people, and police officers. Mobile vans mostly provide a flexible outreach service and act as a bridge to fixed-site outlets. The mobile van can reduce the distance for users to travel to get needles and syringes. Carrying used syringes for long periods in order to exchange presents problems for IDUs in the presence of police pressure and can dissuade them from bringing used syringes back.
Janssen et al. 2009 (Canada)	Cross-sectional	Conducted surveys with 100 women sex workers who accessed MAP services and reviewed MAP logbooks to document use of services. The study assessed the impact of MAP through review of data from a concurrent cohort study of injection drug users and a survey of 97 women at a drop-in center in the Downtown Eastside	Evaluating the impact of MAP (Mobile Access Point van) on safety and adoption of harm-reducing behaviors among sex workers	The number of clean needles dispensed per month almost tripled during the MAP’s first 3 years of operations from 1240 in 2004 to 3241 in 2006. A higher proportion of MAP users were injecting cocaine one or more times daily (31 vs. 19%), but rates of daily heroin injection at about 50% were similar. A higher proportion of MAP users were smoking crack (81 vs. 72%). Rates of borrowing used needles were similar (10%) but none of the MAP users, compared to 10.5% of the non-MAP users, had lent used needles. Over 90% of MAP clients reported that the van made them feel safer on the street. Sixteen percent of surveyed MAP clients recalled a specific incident in which the van’s presence protected them from a physical assault and10% recalled an incident when its presence had prevented a sexual assault.
Kelsall et al. 2001 (Vietnam)	Cross-sectional	*n* = 200; interviews/questionnaires with heroin users who had injected/smoked heroin at least once in the past 6 months	Examining the transitions between different routes of administration and, in particular, transitions between non-injecting—smoking/chasing or “burning”—and injecting routes of administration	Needle/syringe programs (NSPs) were clearly the outlets most favored by the injectors in the sample (76%), including mobile/outreach NSPs (24%). Chemists were cited by injectors as a less common source (28%), as were friends (5%) and community health centers (5%).
Knittel et al. 2010 (USA)	Cross-sectional	*n* = 88; interviews conducted with NEP participants between 2003 and 2006	Evaluating the HARC NEP (run exclusively from the outreach van), describing the operation of the NEP and its clients	Injection-related risk behavior showed non-significant trends in the direction of risk reduction from baseline to follow-up. NEP users at follow-up were less likely to report sharing syringes (OR = 0.66), sharing equipment other than syringes (OR = 0.70), or reusing syringes (OR = 0.34). Follow-up users were also more likely to report exchanging syringes for another individual (OR = 2.77), though this also failed to reach statistical significance. Compared to baseline measurements, NEP participants reused their syringes significantly fewer times before getting new ones (*p* = 0.012)
Lausevic et al. 2015	Cross-sectional	Cross-sectional bio-behavioral survey among PWID	Determining the prevalence of HIV, hepatitis C (HCV), hepatitis B surface antigen (HBsAg), and risk behaviors. HIV in people who use drugs (PWID)	As sources of free-of-charge needles and syringes in the past 12 months, respondents mentioned mobile outreach teams (8.0%), primary health-care centers (17.9%), and drop-in centers (53.5%).
MacNeil and Pauly 2010 (Canada)	Case study	Interviews with clients and NEP staff	This case study focuses on the consequences of the switch to mobile needle exchange services immediately after the closure of a fixed-site needle exchange	The closure of fixed site needle exchange services and the switch to mobile delivery only has had a traumatic effect on clients, with reported increases in risk behavior such as needle reuse as well as a dramatic decrease in access to services. Contacts with vulnerable clients have been lost and thousands of needles are unaccounted for in the community.
Miller et al. 2001 (Norway)	Cross-sectional	*n* = 1260; three consecutive, anonymous cross-sectional surveys. 288, 449 and 523 SEP participants interviewed during comparable 1-week periods in 1992, 1994, and 1997, respectively	Examining gender differences in syringe exchange program (SEP) use, particularly frequent SEP use, within and across survey years. SEP services in Oslo were provided exclusively through one mobile van	During the study week in 1992, 765 recorded SEP visits; in 1994—1348 SEP visits; in 1997, 2175 recorded SEP visits. In 1992 and 1997, women were somewhat more likely to report weekly SEP use than men. During each survey period, just over half of SEP participants reported returning syringes. In 1992 and 1994, women were somewhat more likely to return syringes. Women reported injecting more frequently than men, but neither reported more frequent SEP use nor acquiring more syringes during an exchange. Although syringe sharing decreased significantly over time, in 1997, 51% of SEP participants continued to share. HIV prevalence remained low (3–5%) over time. After controlling for gender, age, and HIV risk factors, frequent SEP use was significantly correlated with frequent injection for both women (OR 5 1.4) and men (OR 5 1.5). A lack of income or benefits independently increased the likelihood of being a frequent SEP user (OR5 3.0), while having shared a syringe at last injection independently decreased this likelihood (OR5 0.5)
Peter 2013 (Nigeria)	Cross-sectional	Two components: harm reduction outreach and a behavioral survey (*n* = 70). For 2 months in the year 2008—provision of mobile base harm reduction services in three neighborhoods	Examining the feasibility and uptake of the night harm reduction services by a late night population of MSM	Exchanged 1090 needles in 121 needle exchange visits, distributed 3200 condoms for both male, and provided 18 HIV tests and 8 opportunistic infections tests. The study population of MSM was characterized by low levels of income, stigma, and discrimination.
Pollack et al. (2002) (USA)	Cohort study	*n* = 373 active IDUs; a pre-post comparison of ED utilization was performed using linked medical records from New Haven’s only two emergency departments	Examining the impact of the New Haven Community Health Care Van (CHCV), a mobile needle exchange-based health care delivery system, in reducing emergency department (ED) use among out-of-treatment injection drug users (IDUs)	CHCV clients included a broad range of extremely disadvantaged individuals. Seventy percent of CHCV clients are unemployed; 35% are current or recent injection drug users. IDUs who obtained CHCV services faced especially high medical and social risks. Twenty-seven percent reported a history of commercial sex work; 26% had been in jail or prison during the 6 months prior to CHCV service use. IDUs seeking services exhibited a mean of 2.9 medical encounters with CHCV staff over the survey period. Among 373 IDUs, 117 (31%) were CHCV clients, and 256 had not used CHCV services. At baseline, CHCV users were more frequent users of ED services (⓪ ` .001). After full-scale implementation, mean ED utilization declined among CHCV clients and increased within the non-CHCV group. CHCV use is associated with statistically significant reductions in ED use. Full-scale implementation of the New Haven Community Health Care Van was associated with a more than 20% decline in emergency department visits
Robles et al. 1998 (Puerto Rico)	Cross-sectional	The data for this study were collected during the first months of the NEP from July 1995 to March 1996 in 13 communities of the San Juan metropolitan area. Subjects were the participants of two modalities of the NEP: a mobile team and a community-based drug treatment program	Evaluating the effectiveness of the first needle exchange program (NEP) established in Puerto Rico	The mobile unit reported 10,770 exchange contacts with 93,066 syringes exchanged; the on-site modality reported 8425 exchange contacts and 53,257 syringes exchanged. More women participated in the on-site modality (22.8%) than the mobile modality (16.3%) (Pf0.01). There were no significant differences between the two modalities in years of injection (11.5 years in the mobile vs 12.1 years in the on-site modality; Pf0.27). However, participants in the on-site modality were more likely to inject more frequently than in the mobile modality (7.2 vs 5.9, Pf0.01).
Rose et al. 2006 (USA)	Cross-sectional	The study had two components: harm reduction outreach and a behavioral survey. For 4 months during 2004, we provided van-based harm reduction services in three neighborhoods in San Francisco from 1 to 5 a.m. for anyone out late at night. We also administered a behavioral risk and service utilization survey among MSM. *n* = 55	The purpose of the Late Night Breakfast Buffet (LNBB) (mobile service) was to determine the feasibility and uptake of harm reduction services by a late night population of MSM. The “buffet” of services included: needle exchange, harm reduction information, oral HIV testing, and urine-based sexually transmitted infection (STI) testing accompanied by counseling and consent procedures	Exchanged 2000 needles in 233 needle exchange visits, and 200 packages containing 3 sterile syringes were provided to individuals who had no syringes to exchange, distributed 4500 condoms/ lubricants and provided 21 HIV tests and 12 STI tests. Of the 36 MSM who reported ever injecting, 75% reported using a needle exchange service. Van-based mobile outreach unit reached a disenfranchised population of MA-using MSM who are at risk for acquiring or transmitting HIV infection through multiple high-risk behaviors, and we established the feasibility and acceptability of late night harm reduction for MSM and MSM who inject drugs. The LNBB corroborated earlier findings of a larger seroprevalence study among a similar population and established an effective methodology for reaching a high-risk population of MA-using MSM, half of whom were injection drug users (IDUs).
Schwartz 1993 (USA)	Review	Overview of the first large-scale syringe and needle exchange (SANE) programs		During the first 7.5 months of the program’s operation, 700 IVDU were enrolled (mean age 34, only 8% less than 25). 60% seropositive for HIV. Approximately equal numbers of white, black, and Hispanic clients. Approx. 25 new clients enrolled each week. In the first 7.5 months, 275 of clients were placed in treatment programs, mainly MMT and detoxification programs. The rate of return injection equipment was 52%.
Shannon et al. 2008 (Canada)	Cross-sectional	*n* = 198; interview-questionnaires, administered by trained peer researchers, with 198 women in street-level sex work in Vancouver, Canada	Exploring how health service and syringe availability may be impacted at the geographic level by avoidance of physical settings due to violence and policing among women in street-level sex work	Approximately half of current female IDUs (56%) had accessed syringes from a fixed site (56%) (including hotel exchange, pharmacy, and clinic) and the medically supervised injection facility (47%). In terms of mobile resources, 50 (43%) of women had accessed syringes from a mobile van in the core area, 20 (17%) from a mobile van in either of core or perimeter areas, and 13 (11%) from outreach workers, while 59 (29%) had accessed a mobile van for other harm reduction resources and referral in either of core or perimeter areas.
Somlai et al. 1999 (USA)	Qualitative	Ethnographic field observations and key informant and systems representative interviews	Illustrating how social science and community assessment research can be used to guide the development of NEPs.	Mobile van unit was found to be more acceptable alternative to a fixed site by the IDU’s and community members in one of Milwaukee’s neighborhoods.
Spittal et al. 2004 (USA)	Qualitative	Ethnographic interviews and observations conducted with fixed site and mobile van “exchange agents”	Examining the access to sterile syringes by IDUs in Vancouver between May 2000 and March 2001	Interviews and observations suggest that on any given van route, the “loaners” make up roughly 5–10% of the syringes distributed to clients. The demand for “loaners” was greatest along the mobile routes, especially at particular stops within the Downtown Eastside where numerous clients appeared with nothing to exchange. Clients visiting the mobile exchanges are often low-volume, high-frequency traders. These clients may not have other avenues through which to access needles, especially through the evening and into the night.
Stark et al. 1995 (Germany)	Cross-sectional	Participants (*n* = 557) were recruited from drug-free long-term treatment centers, a storefront agency and a syringe exchange bus	Investigating differences in prevalence and determinants of HIV infection, and in recent risk behavior (previous 6 months) among injecting drug users (IDUs) who are in contact with different types of services for IDUs in Berlin	Participants entering long-term treatment were most likely, and IDUs at the syringe exchange bus were least likely to have borrowed and passed on syringes in the previous 6 months. Of the individuals examined at the storefront agency and at the syringe exchange bus, 20.7 and 14.6% were HlV-infected, respectively, HBV and HCV seroprevalence rates were significantly lower among IDUs entering treatment (*n* = 157) than among IDUs at the storefront agency (*n* = 203) (anti-HBc 48.4 vs 63.5%, *p* < 0,005; anti-HCV 73.1 vs 89.8%, *p* < 0.0001). IDUs entering treatment had injected for a significantly shorter time: their median time since first injection was 6 years (interquartile range 3–11), compared to 11 (5–16) years (storefront agency), and 9 (5–15) years (syringe exchange bus) (*p* < 0.0001)
Strathdee et al. 2006 (USA)	RCT	*n* = 245 IDUs; a randomized trial of a case management (intervention) versus passive referral (control) among NEP attenders requesting and receiving referrals to subsidized, publicly funded opiate agonist treatment programs	Evaluating a case management intervention to increase treatment entry among injecting drug users referred from 2 mobile needle exchange programs (NEP)	Those who were randomized to case management were more likely to enter treatment within 7 days. Additional “as treated” analyses revealed that participants who received 30 min or more of case management within 7 days were 33% more likely to enter treatment and the active ingredient of case management activities was provision of transportation.
Strike et al. 2002 (Canada)	Qualitative	Using a modified ethnographic approach (i.e., interviews and observations), NEP staff and managers at all Ontario NEPs and government officials involved with the Ontario provincial needle exchange program participated in semi structured, audio-taped interviews (11/98 to 04/99)	Examining the challenges of four service delivery models (i.e., fixed, mobile, satellite and home visits) and how service delivery may impact on NEP HIV prevention efforts	Mobile service is believed to increase accessibility for clients who prefer to exchange during evening hours, do not have a vehicle, money for transportation, and/or may be too impaired to drive to the fixed site. While mobile service is believed to meet the needs of clients in terms of basic services, it is viewed as insufficient for lengthy counseling sessions, arranging referrals, HIV, and other disease testing
Tinsman et al. 2001 (USA)	Cross-sectional	*N* = 9296; clients were recruited through 12 HIV Outreach Project sites	Describing 12 HIV Outreach Demonstration Project and summarizing the findings of multivariate statistical analyses aimed at identifying important project and client characteristics that influenced project success	Projects used multiple outreach strategies to attract the “hard-to-reach” population. The projects that used mobile testing units to reach their clients were vastly more successful than other projects in their HIV testing efforts: all else being equal, outreach clients at projects with mobile units were 86 times more likely to obtain an HIV test than those at other outreach projects. Mobile units or vans provided a movable center for outreach services such as counseling and testing as well as transport for outreach workers to target neighborhoods. Advantages to mobile units included high community recognition of the outreach project, safer place for worker/contact interaction, and relative privacy for outreach contacts and service provision. They also served as a focal point to initiate conversations.
Wood et al. 2002 (Canada)	Cohort	*N* = 761: persons who returned for follow-up during the period June 1, 2000/May 31, 2001; active drug injectors	Providing possible explanations for persistent needle sharing through an evaluation of the Vancouver Injection Drug Users Study (VIDUS), an ongoing cohort study of IDU that began in 1996	351 (46.1%) of participants acquired most of their needles from the fixed site exchanges, 109 (14.3%) from the exchange vans, 60 (7.9%) from pharmacies, and 241 (31.7%) acquired needles from multiple sources and did not identify a primary source. The present study suggests that difficulty meeting the exchange van poses problems and shows that exchange vans may be an effective means of providing services to IDUs in areas of low injection drug use prevalence and not being served by a fixed-site.

### Service delivery model characteristics

From all the publications that met inclusion criteria, nine studies examined the service delivery characteristics and these originated from USA, Canada, and Russia. Mobile NSPs typically operate from vans and buses [16, 17]. In addition to distribution and disposal of injection related equipment (e.g., needles/syringes, cookers, swabs, and/or biohazard bins), reports show that some mobile NSPs offer harm reduction education, HIV testing and counselling, urine screening for sexually transmitted infections, distribution of equipment to smoke drugs (e.g., crack kits), emergency medical help, peer counseling, condom distribution, drug treatment information and referrals, referrals to other services (e.g., reproductive health), methadone maintenance treatment, and supervised injection services [18–25].

Space was noted to limit the total number of services that can be offered on board and also create impediments to client privacy when they access mobile NSP services [26]. A study in 2010 by MacNeil and Pauly examined the changes in services after a mobile NSP was introduced following the forced closure of a fixed site NSP [27]. The mobile NSP was reported to have maintained contact with the clients and provided basic services but was insufficient to compensate for the full range of service previously offered by the fixed site. Contacts with clients through mobile NSP were of shorter duration and mainly reduced to providing new needles. For that reason, most clients advocated for operation of both fixed and mobile site NSP [27].

Mobile NSPs are designed to serve clients in varied locations and often at times when NSP services may not be available. Mobile outreach units can cover a large geographic area, often follow predictable routes, and make stops at prearranged times when clients are most likely to access services. In Volgograd, Russia, a mobile van covered three networks of drug users during the 40-km stretch along the Volga River in addition to a fixed-site NSP [28]. As well as stopping at public locations, some mobile NSPs respond to client telephone requests for delivery of equipment to their homes and through secondary peer distribution [26]. Strike and colleagues (2002) note that through home delivery mobile NSPs in Ontario, Canada, were able to serve clients with disabilities and those who did not want to access fixed-site NSP [26].

Studies report that mobile NSPs can adapt service delivery to changes in local drug scene and incorporate additional populations of people who use drugs better than do other models of NSP service delivery [8, 17, 29]. A study from Vancouver showed [30] that when cocaine became the predominant drug used for injecting, demand for needles increased and the number of mobile NSPs was increased from one to three to meet the rising demand for injecting equipment.

In locations with high police surveillance, mobile NSPs can make accessing services less identifying for clients [17]. Tinsman et al. [31] examined 12 outreach projects in the USA and found that mobile NSP projects were highly recognized by the local community of people who use drugs, provided a safer and relatively private environment for interaction between clients and staff to receive harm reduction services and HIV testing. Conversely, mobile NSPs were noted to be less recognizable and to attract less attention from neighbors, local businesses, and the police [17]. Somlai et al. [32] reported that mobile NSPs are more acceptable to community residents who fear that a fixed site will draw violent and criminal behavior to the neighborhood.

Although mobile NSPs can visit several locations a day, reports note that services are often provided for limited amounts of time and may be a barrier to accessing clean needles [27, 29, 30, 33]. Some people who use drugs might not be able to access services during those times and may not be covered by through mobile outreach [29]. Hyshka et al. [30] found that the mobile van was the only outlet providing needles during evening hours in Vancouver and many people who use drugs were not covered either by the fixed-site or mobile program. Wood and colleagues [33] suggested that “missing the van” could be considered as a risk factor for high-risk needle sharing since needle sharing was 3.5 times higher in the population of users who had difficulty accessing new needles through the van compared to those who did not have difficulty.

### Client characteristics

A total of 14 studies examined the characteristics of the clients who accessed mobile NSP services. These studies originated from the USA (including Puerto Rico), Ireland, Canada, France, Nigeria, and Germany. While the design, objectives, and measured outcomes varied, all 14 studies confirmed that mobile NSP units attract people who use drugs and report high-risk injection behaviors [5, 6, 8, 17–20, 34–39] and also populations not covered by other service models [17–21, 33, 39–48]. Mobile NSPs were noted to increase access for “hard-to-reach populations” by removing the barriers such as fear of public exposure occasioned by entering a fixed site, stigma, mobility issues associated with physical disability, local policing practices that discourage attendance at fixed sites, and poor availability of public transportation services [49].

Specifically, mobile NSPs were shown to attract people who are under the age of 18 [41], homeless [17], out-of-drug-treatment [42], men who have sex with men and who use methamphetamines [19, 20], women involved in street-level sex work [18, 22, 39, 43], drug-using ex-offenders [21], and the Roma population [44]. In San Francisco [19] and Nigeria [20], the mobile NSPs were designed to reach men who have sex with men and who inject drugs. Courty [41] reported that clients attending mobile NSP bus at Clermont Ferrand, France, were predominantly younger (under the age of 18), had limited information regarding various health issues, and were not in contact with other health and service providers.

Studies show mixed results in terms of the gender of the clients reached by mobile NSPs. Allen et al. [50] reported that the majority of participants (71%) accessing the largest mobile NSP in Washington, USA, were male. Conversely, a study from Vancouver reported that the mobile NSP served a higher percentage of female clients [17]. A study from Puerto Rico found that clients who accessed mobile NSP were less likely to be female compared to a fixed site [35]. In Oslo, Miller et al. [51] found that in 1992 and 1997, women were somewhat more likely than men to report weekly use of the mobile NSP in Oslo, Norway.

Studies also show that mobile NSPs tend to attract high frequency and higher risk injectors than fixed site or pharmacy-based programs [6, 8]. Several studies reported that the frequency of injection was high among the clients accessing mobile NSP [5, 8, 17, 19, 29, 37, 38, 52]. In Oslo, Norway, clients used more needles each day than clients attending other service models [29]. In other studies, results show mobile NSPs attracted populations of high-risk cocaine injectors [8, 18, 39]. Conversely, a study in Puerto Rico, by Robles et al. [35] found that clients of the fixed sites reported a higher frequency of injecting than clients who used the mobile service.

Riley et al. found that clients accessing van-based outreach services in Baltimore were more likely to be cocaine injectors, to have injected with previously used needles, and inject more frequently than clients who accessed services at pharmacies [8]. In Vancouver, sex workers who accessed van-based services provided by the Mobile Access Point were found to be more likely to have injected cocaine one or more times daily in the last 6 months than sex workers who were clients of the drop-in center at the Downtown Eastside, Vancouver [18]. Rose et al. [19] found that high rates of methamphetamine use among clients accessing mobile van providing harm reduction services including NSP to the late night population of men who have sex with men (MSM) in San Francisco. Of all MSM clients, the majority of MSM (97%) injected methamphetamine and 13% injected it several times a day. Methamphetamine use and alcohol consumption were noted as risk factors for having unprotected sex among HIV-negative and HIV-positive MSM [19].

Needle sharing, borrowing, and re-use were found to be higher among the population of clients reached by mobile NSPs [8, 53]. However, a study conducted in Berlin showed that clients of the mobile NSP were less likely to have shared and borrowed needles in the last 6 months than clients using other service models [34].

Two studies reported that the mobile NSP served sex workers who tended to have more clients on average in the last week, to provide sex services to clients in alleys, industrial sites, and an outdoor setting, compared to clients who did not use mobile NSP services [18, 39]. A study from 2008 [43], showed that although the majority of female sex workers who use drugs (56%) obtained needles from a fixed site, a mobile NSP unit was an important venue for accessing needles and other harm reduction services in several different locations in Vancouver. Specifically, 43% of women had accessed needles from a mobile NSP in the core area, 17% from a mobile NSP in either of core or perimeter areas, and 11% from outreach workers, while 29% had accessed a mobile NSP for other harm reduction resources and referral in either of core or perimeter areas. From a survey of 100 women in a drop-in center in Downtown Eastside in Vancouver, Janssen et al. [18] reported that 81% of women had accessed services in the Mobile Access Point (MAP), a peer-led mobile NSP unit that provided emergency medical help, peer counseling, condoms, and new needles, resource information, and referral. The majority of women (44.8%) reported accessing MAP services once each night, and the remainder of women accessed services twice each night (20.9%), twice weekly (17.9%), once weekly (11.9%), or less frequently (3.0%) [18]. In 2006, an average of 1496 women accessed the MAP van per month [39].

### Service utilization data

A total of 10 studies reported service utilization data and these originated from the USA, Canada, Nigeria, Germany, Norway, Lithuania, and Montenegro. The New Haven Needle Exchange mobile van reached 700 clients in the first 7 months of operation. Of these, 60% were seropositive for HIV [54]. In San Francisco [19], a mobile unit exchanged 2000 needles in 233 exchange visits over a 4-month period in 2004. A study assessing the percentage of the at-risk population reached by mobile NSP in two US cities, New Haven and Chicago [38], found that 100,000 needles were distributed between November 1990 and October 1993 by a mobile NSP in New Haven. In 2004, a mobile NSP in Chicago reached an average of 1000 clients every month [38]. Janssen et al. reported that the number of needles dispensed through the Vancouver Mobile Access Project tripled during the first 3 years of its operation, from 1240 in 2004 to 3241 in 2006. An average of 1496 clients per month accessed MAP van in 2006 [18]. In Nigeria, a mobile NSP project exchanged 1090 needles across 121 visits between July and September 2007 [20]. The only existing NSP program in Oslo [51], which operated from a mobile van showed that the number of visits tripled between 1992 and 1997 (i.e., 765 visits in 1992, 1348 in 1994, and 2175 in 1997). The “Blue Bus” exchange project started in 2001 in Vilnius, Lithuania, and it recorded high numbers of distributed needles in the first months of operation [44]. An already existing outreach program that operated without a mobile unit in Vilnius distributed 16,700 needles and 17,350 needles in the first year of its operation (1997), and with the addition of a mobile NSP, the number of distributed equipment increased significantly: 56,000 syringes and 64,580 needles distributed in 2001, and 73,408 syringes and 87,733 needles in 2002. Within 1 month from the beginning of the operations of the “Blue Bus,” 350 clients were using its services. The “Blue Bus” project in Vilnius was able to reach 40% of the city’s population of people who inject drugs. Fischer [55] stated that mobile NSP programs in Frankfurt were the major source for needle distribution, providing 80% of all needles in the city.

In Podgorica, Montenegro, 8% of clients reported using a mobile NSP to access new needles in the last 12 months but many more reported accessing these supplies at primary health-care centers (17.9%) and drop-in centers (53.5%) [56]. In Vietnam, Kelsall et al. [57] found that 76% of clients reported using NSP as a preferred outlet for accessing needles, including mobile/outreach NSPs (24%).

Some mobile NSPs also collect used equipment for disposal [51, 54]. Indeed, in New Haven, Connecticut, Schwartz reported that 52% of used injecting equipment was collected by the mobile van [54]. In Oslo, Norway, rate of returned needles was over 50% during the three survey periods of the study—1992, 1994, and 1997—and women were found to be more likely to return needles for the first two survey periods [51].

### Referral of clients to other services

We identified eight studies from the USA, Canada, Germany, and France, which reported on efforts to refer clients to services which were not available directly through mobile NSP [5, 31, 39, 41, 42, 54]. Schwartz [54] found that within 7.5 months of operation, 275 clients of the mobile NSP in New Haven, Connecticut, were admitted to drug treatment programs (i.e., most methadone maintenance) through referrals offer by van staff. The bus operating in Clermont Ferrand, France, referred young people who injected drugs to health and social services that previously had not been accessed [41]. The CHCV van in Baltimore found that 64% of the clients who received HAART treatment through a mobile NSP also were successfully referred to and entered into drug treatment in the 12-month follow-up period [42]. In Vancouver, a mobile NSP that served a high-risk population of sex workers was found to have an important role in facilitating entry and increasing utilization of residential and detoxification treatment. During the 18-month period of the study, accessing inpatient addiction treatment was reported 45 times and outpatient treatment was reported 162 times. Accessing the mobile NSP was significantly associated with the utilization of inpatient addiction treatment [39]. Similarly, a project in Ypsilanti, Michigan [53], where NSP was provided through a HIV/AIDS Resource Center (HARC) Harm Reduction mobile van, showed between 2003 and 2006 the clients in the follow-up period (6 months after they started using van services) were more likely to enter drug treatment compared to the baseline group.

### Specialized interventions

A total of three studies conducted in USA and Canada explored the impact of specialized interventions delivered through mobile NSPs. Pollack et al. [36] found that the Community Health Care Van (CHCV) in New Haven, a mobile-based NSP and health care delivery system, was effective in reducing the rates of emergency department use among out-of-treatment injection drug users. The van, equipped with two examination and one counselling room, provided clinical care, diagnosis, and treatment of tuberculosis and syphilis vaccination and general health education. Even though 35% of the clients who used CHCV services faced high medical and social risk, full-scale implementation of the CHCV van services resulted in a more than 20% decline in emergency department visits. Altice and colleagues [42] reported on a pilot project offering HIV treatment and also HIV counseling, case management, drug treatment coordination, health status assessment, and acute and episodic medical care on a mobile NSP. This pilot project involved the delivery of HAART treatment through the CHCV van to active heroin injectors at the mobile NSP locations. Through this pilot project, 13 clients initiated antiretroviral therapy in the mobile NSP and of these, 9 (69%) entered drug treatment once they achieved non-detectable viral load. The authors suggested that the delivery of HAART therapy through mobile NSP can improve access to HAART treatment for the population of out-of-treatment HIV-positive drug users who are already using mobile NSP services.

A randomized control trial in Baltimore [58], showed that provision of case management through a mobile NSP was successful to facilitate referrals for entry into drug treatment. The authors reported that NSP clients who were randomized to case management were more likely to enter drug treatment within 7 days than the clients who received only a passive referral to treatment. The case management group also received transportation to treatment settings suggesting that offering transportation may reduce barriers to treatment for disadvantaged populations of people who use drugs [58].

### Impact of mobile NSPs on injection risk behaviors

Only two studies reported the impact of mobile NSP on rates of needle sharing. A study in Oslo found a significant decrease in past year needle sharing from 64% of clients in 1992 to 51% in 1997 [51]. In a study by Knittel et al. [53], NSP staff in Ypsilanti, Michigan, interviewed clients at intake and after 6 months of accessing the HARC NEP that operated 3 days a week from a van. After 6 months of using HARC mobile NEP services, clients were less likely to share needles (OR = 0.66), share injecting equipment other than needles (OR = 0.34), and reuse needles (OR = 0.34) compared to baseline reports. Although the study collected data at only one point in time, Subata and Kriksciukaityte [44] noted that 96% of clients did not re-use needles and 88% did not share needles in the last 30 days and attributed these rates to use of the mobile NSP in Vilnius.

## Discussion

Riley et al. recommend implementation of several NSP service delivery models to offset the limitations of particular models and improve access for clients [8]. In terms of contribution to NSP goals, results from this scoping study show that mobile NSPs:

- Have been implemented across the world in high-, medium-, and low-income countries
- In addition to distributing injection-related equipment, provide a wide range of other harm reduction, health, and social services
- Face limitations to service complement, confidentiality, and duration of interactions imposed by physical space
- May shield program utilization from police observation
- Can adapt to changes in locations and types of drug use
- Attract people who engage in high-risk and high-intensity injection behaviors
- Attract clients with diverse social characteristics and who are often not reached by other service models
- Provide referrals to varied health and addiction services
- Can offer specialized interventions onboard (e.g., primary care, HIV treatment, case management)
- May lead to reduced injection-related risks

It is not clear from the literature reviewed, however, what are, or if there are, a “core and essential” complement of services that mobile NSPs should offer. Decisions about service complement for mobile NSPs need to be made in relation to the context and also other available services. The WHO, UNODC, UNAIDS technical guide [11] provide guidance regarding essential HIV prevention services and in addition to NSP services includes on the list services that some mobile NSPs do offer (i.e., opioid substitution therapy; HIV testing and counselling; antiretroviral therapy; prevention and treatment of sexually transmitted infections; condom distribution; targeted information, education and communication and prevention, vaccination, diagnosis and treatment for viral hepatitis). As well, the WHO recommends a variety of ways to make services user friendly that are directly addressed by mobile NSPs (e.g., co-location of interventions, strategic location of services in “hotspots”) [11].

Reports of client visits to mobile NSP provide a picture of the volume and frequency of utilization but are difficult to compare given varied measures and reference periods. Measures of program coverage and reach [59] might provide clearer metrics to assess mobile NSPs as standalone interventions, as part of an NSP offering varied services models and in relation to larger HIV prevention activities. However it is important to highlight that although member states at the UN General Assembly Special Session on Drugs endorsed NSPs as one of several programs to reduce adverse public health consequences of drug abuse, a recent report from Harm Reduction International noted that NSP coverage across the world is below the level required to prevent HIV and HCV among people who inject drugs.

Our scoping study is not without its limitations. First, we searched the published, English literature for studies reporting empirical literature about mobile NSPs. As such, non-English and grey literature is not included in our summary of the extant literature and may not reflect all that is known across the globe about mobile NSPs. However, given time and budget constraints, we did not search the grey literature which is vast and with potential reports embedded and repeated within and across many websites. In addition, if there had been resources for a search of the grey literature, we would have had to assess but may not have had sufficient information to determine if the methods used to collect the data supported the findings reported. Second, this scoping study includes and disseminates findings from a range of studies utilizing different methods and study designs providing narrative or descriptive accounts of available empirical research but does not provide a quality appraisal needed for a systematic review. This is typical for scoping studies. Given the heterogeneity of the study designs and data collected reported in this manuscript, we did not proceed with a systematic review. Third, the two authors met and agreed on the search strategy, inclusion, and exclusion criteria; reviewed each abstract; and reached consensus on the organization, extraction, and charting of the data. It could be that others may organize the data differently, but we believe our presentation of the findings is faithful to the original studies.

## Conclusion

Mobile NSPs have an important role to play in improving HIV and HCV prevention efforts across the world. However, more work is needed to create clearer assessment metrics and to improve access to NSP services across the world.

## Abbreviations

HCV: Hepatitis C
HIV: Human immunodeficiency virus
NSP: Needle and syringe programs
